# Axl as a mediator of cellular growth and survival

**DOI:** 10.18632/oncotarget.2422

**Published:** 2014-11-11

**Authors:** Haley Axelrod, Kenneth J. Pienta

**Affiliations:** ^1^ The Cellular and Molecular Medicine Program, The Johns Hopkins School of Medicine, Baltimore, MD, USA; ^2^ The James Buchanan Brady Urological Institute, Department of Urology, The Johns Hopkins School of Medicine, Baltimore, MD, USA; ^3^ Department of Oncology, The Johns Hopkins School of Medicine, Baltimore, MD, USA; ^4^ Department of Pharmacology and Molecular Sciences, The Johns Hopkins School of Medicine, Baltimore, MD, USA; ^5^ Department of Chemical and Biomolecular Engineering, Johns Hopkins University, Baltimore, MD, USA

**Keywords:** Axl, TAM receptors, Gas6, cancer, tyrosine kinase, proliferation, apoptosis, immune, migration, inhibitor

## Abstract

The control of cellular growth and proliferation is key to the maintenance of homeostasis. Survival, proliferation, and arrest are regulated, in part, by Growth Arrest Specific 6 (Gas6) through binding to members of the TAM receptor tyrosine kinase family. Activation of the TAM receptors leads to downstream signaling through common kinases, but the exact mechanism within each cellular context varies and remains to be completely elucidated. Deregulation of the TAM family, due to its central role in mediating cellular proliferation, has been implicated in multiple diseases. *Axl* was cloned as the first TAM receptor in a search for genes involved in the progression of chronic to acute-phase leukemia, and has since been established as playing a critical role in the progression of cancer. The oncogenic nature of Axl is demonstrated through its activation of signaling pathways involved in proliferation, migration, inhibition of apoptosis, and therapeutic resistance. Despite its recent discovery, significant progress has been made in the development of effective clinical therapeutics targeting Axl. In order to accurately define the role of Axl in normal and diseased processes, it must be analyzed in a cell type-specific context.

## The TAM receptor tyrosine kinase family mediates the function of protein S and Gas6

**Figure 1:**
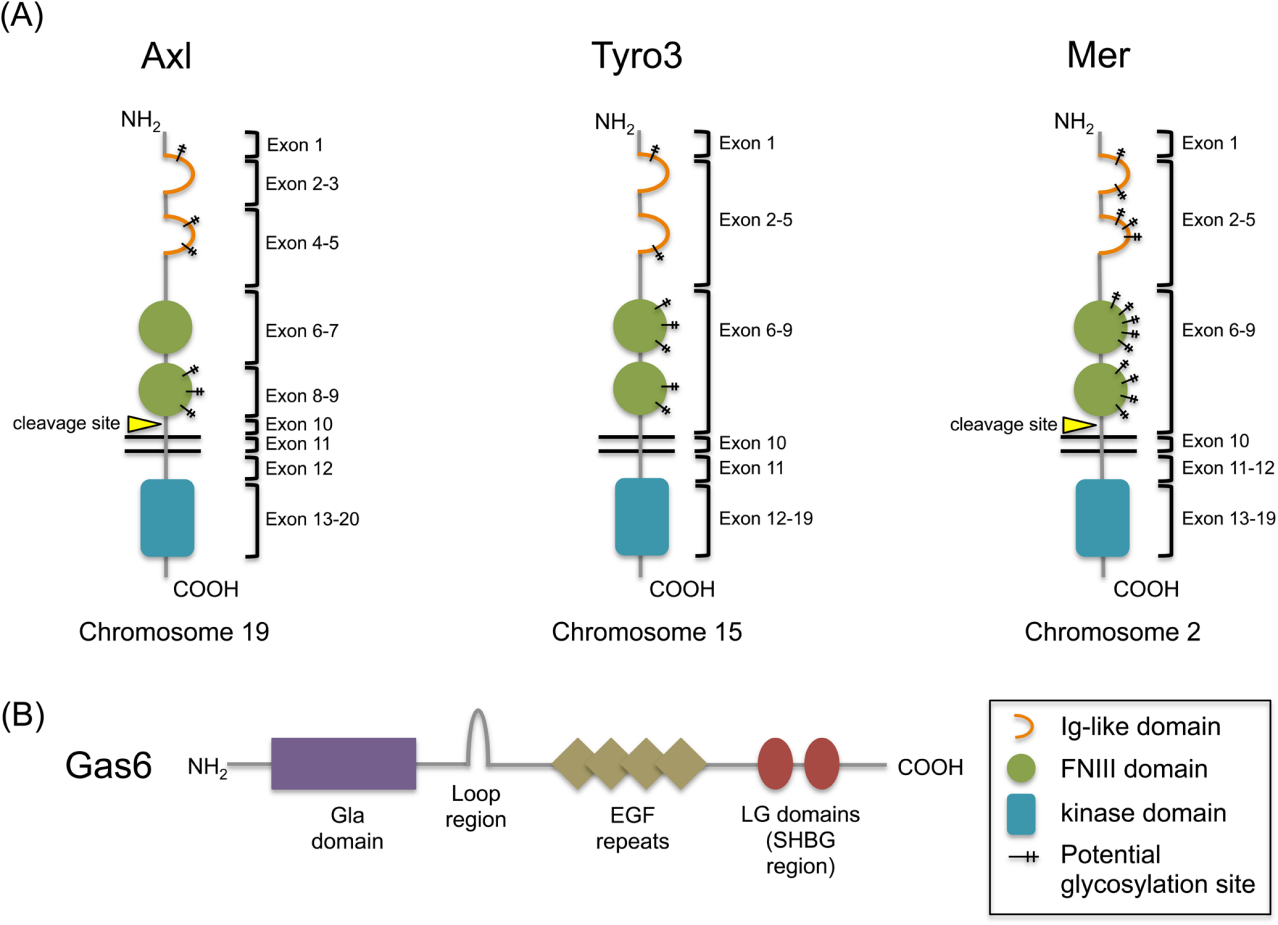
Structures of the TAM receptors and their shared ligand, Gas6 **(A)** The TAM family of receptors share common extracellular structures, composed of two Ig-like domains for ligand binding and two fibronectin III domains. Axl and Mer have both been shown to yield soluble extracellular fragments by protease cleavage just outside their transmembrane domains. To date, this has not been demonstrated for Tyro3. Potential glycosylation sites are represented on each receptor; Axl, amino acids 43, 157, 198, 339, 345, 401; Tyro3, amino acids 63, 191, 230, 240, 293, 366, 380; Mer, amino acids 114, 170, 207, 215, 234, 294, 316, 329, 336, 354, 389, 395, 442 (confirmed), 454. **(B)** Gas6 is a vitamin K-dependent protein that binds Axl with higher affinity compared to Tyro3 or Mer. The Gla domain allows for cell membrane contact and the LG domains bind the Ig-like domains of the receptors.

Receptor tyrosine kinases (RTKs) are classified into families based on their structural and functional properties. The TAM (Tyro3, Axl, Mer) family is set apart based on a unique extracellular domain and common ligands (Figure 1). As a Type I receptor family, their N-termini are decorated by pairs of immunoglobulin (Ig)-like and fibronectin III (FNIII) domains. At the time of their discovery, this pattern had not been observed in other RTKs. In 1991 *Axl* was cloned as the first TAM receptor in which this pattern was observed, and subsequent cloning of both *Tyro3* and *Mer* in 1994 revealed the existence of similar domains [1–3].

The TAM receptors are also grouped based on their common ligands, protein S and Gas6. While Gas6 is able to bind all three TAM receptors, however, protein S is only able to bind Tyro3 and Mer [4]. Although there is some confusion in the literature regarding this finding, it may be due to the absence of a pattern of charged residues in protein S, which help form the major contact of the Gas6/Axl interaction [4–6]. Both ligands share 44% amino acid identity and are both vitamin K-dependent, owing to their shared γ-carboxyglutamic acid (Gla) domain. The Gla domain allows for cell membrane contact through calcium-dependent phospholipid binding, and is present in all vitamin K-dependent proteins [7]. Carboxylation of Gas6 and protein S is necessary for their activation of the TAM receptors, and thus inhibitors of vitamin K such as warfarin are able to block TAM receptor signaling, indicating a further level of control [8–11]. Protein S and Gas6 also share a region of homology in their four EGF-like domains, which mediate cell-cell communication [12, 13]. Physiologic differences between the two proteins are that Gas6 is present at ~0.2nM in human plasma and is complexed with the soluble form of Axl, whereas protein S is present at 1,000 times higher concentration and 60–70% is bound to the complement regulator C4b-binding protein (C4BP) [14–16]. Initially, it was thought that protein S was the ligand for Tyro3 (Sky, BYK, Dtk, RSE, Tif), Gas6 was the ligand for Axl (Ufo, JTK11), and that additional protein S-related factors were potential candidates as the ligand for Mer (c-mer, RP38) [17]. By the time these were established as activating ligands, protein S had already been functionally characterized as a negative regulator of the coagulation pathway. However, the function of Gas6 was unknown. Later studies have shown that Gas6 is actually a common ligand for all three receptors, having the highest affinity for Axl, followed by intermediate and minor affinities for Tyro3 and Mer, respectively [18]. The Gas6 gene was cloned in 1988 and characterized in 1993. Its name derives from its discovery – in a hunt for regulators of cell cycle arrest, Schneider et al. termed their findings “growth arrest-specific” factors [19]. The origin of Gas6 suggests a functional role for the TAM receptors in protection from cell death, and indeed later studies have proposed various roles for the receptor family in cell survival. Furthermore, the roles of Axl, Tyro3, and Mer extend to mediation of processes such as proliferation, migration, and adhesion in both normal and disease settings. The signaling overlap downstream of these receptors is evidence of their functional similarities, however much of the context- and receptor-specific signaling remains uncertain. Indeed, it is important to note that these roles are cell context-dependent, highlighting their complexity.

## Discovery of Axl

*Axl* was first isolated as an unidentified gene detected in two separate patients when Liu et al. began a search for transformants in chronic myelogenous leukemia in 1988 [20]. A few years later it was characterized and given the name “Axl,” derived from the Greek term “anexelekto,” or uncontrolled, based on the initial observations of its function [2]. Coincidentally in 1991, Janssen et al. cloned the same gene from a patient with a chronic myeloproliferative disorder, which they termed “UFO” for its unknown function [21]. The gene was shown to have low transforming potential that was not enhanced after multiple passages. When Axl was characterized in 1991, it was found that it was necessary but not sufficient for transformation [2, 20]. Rather, the transforming property of Axl was suggested to be due to a selection of its overexpression in cells, and to date, there have not been any activating mutations found [2, 22].

## The structure of Axl

The *Axl* gene is located on the long arm of chromosome 19, at position q13.2 [2]. The protein is approximately 140kDa in the fully glycosylated state, and is 120kDa when partially glycosylated (Figure 1). There are long and short isoforms of *Axl*, differing in the presence or absence of exon 10 by alternative splicing; the full-length isoform is the most abundant [2]. The alternative splicing of *Axl* has not been well studied, but it is proposed that inhibition of PKC coupled to downstream splicing effectors can induce exon skipping [23].

### Exon 1

The insertion of Axl into the plasma membrane is dependent upon the presence of a signal peptide located in exon 1 (Figure 1).

### Exons 2–5

**Figure 2:**
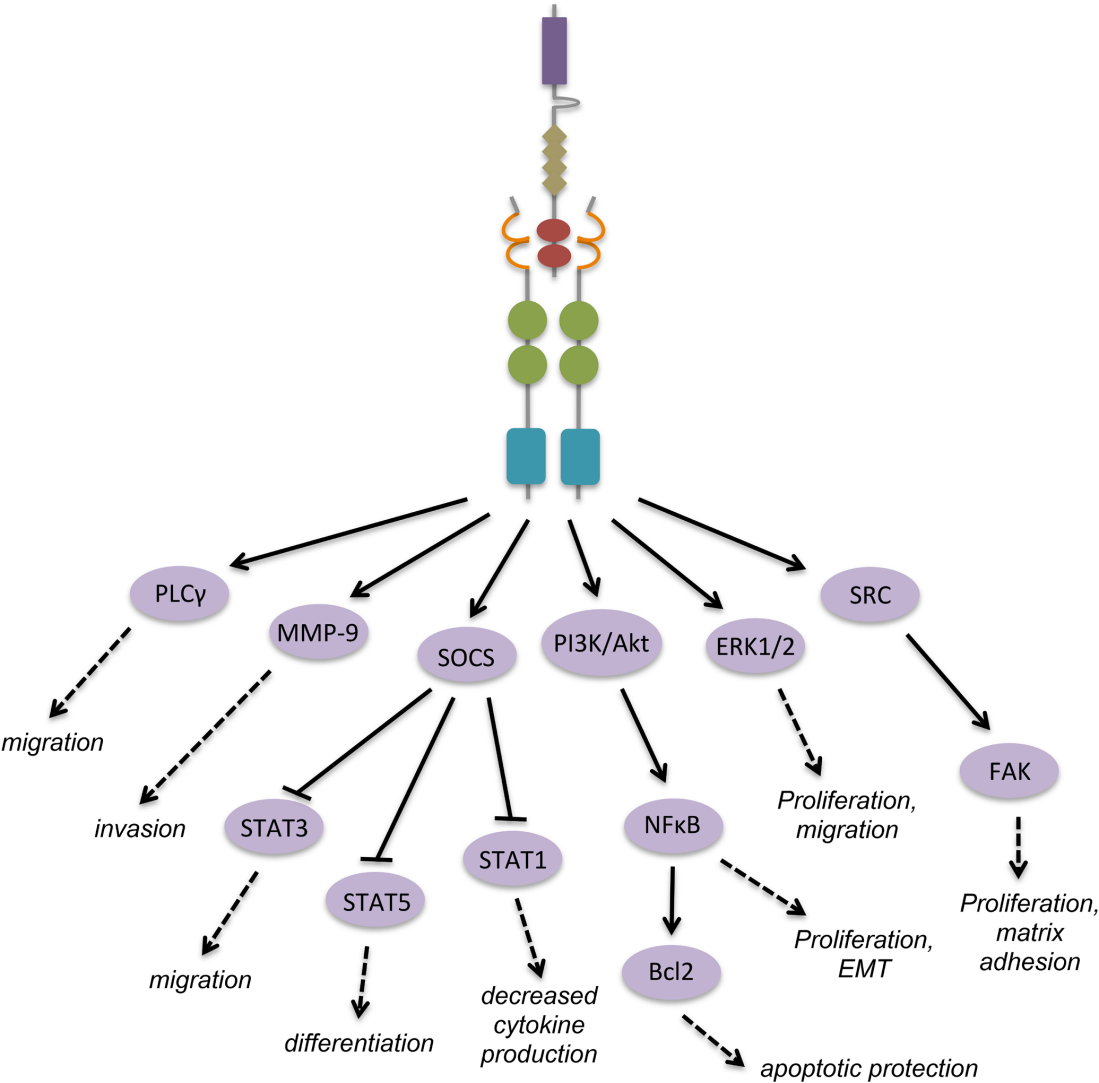
Gas6 activation of Axl leads to homodimerization and activation of downstream signal cascades with functional consequences The above signaling diagram represents events downstream of Gas6 binding and Axl homodimerization across many cell types. Gas6 binding to Axl creates a major contact formed between the LG1 domain of Gas6 and the Ig-like 1 domain of Axl, and a minor contact between the LG1 domain of Gas6 and the Ig-like 2 domain of Axl. Other ligands not shown: protein S contains the same domains as Gas6, and binds Tyro3 and Mer through its SHGB region; tubby and Tulp1 contain “minimal phagocytic determinants” (MPDs) in their N-termini which are essential for receptor binding; tubby binds Mer and Tulp1 binds all three TAM receptors.

Exons 2–5 make up the two Ig-like domains, which bind one laminin G-like (LG) domain in the sex hormone-binding globulin (SHBG) region of Gas6 (Figure 1). The structure of the Axl-Gas6 complex reveals that the first and second Ig-like domains of Axl form the major and minor contacts, respectively, with only the first laminin G-like domain in Gas6 (Figure 2) [24]. Binding in this manner prevents any direct Axl/Axl or Gas6/Gas6 contacts. The minor contact is conserved in Tyro3 and Mer, however the major contact responsible for high affinity binding is not, reflecting the hierarchy of Gas6 binding to each of the receptors [24].

### Exons 6–9

The FNIII domains in exons 6–9 provide the basis for the proposed role of Axl in adhesion (Figure 1). These domains are found within other adhesion molecules such as the neural cell adhesion molecule (NCAM), and fibronectin itself acts as molecular bridge for integrins and extracellular matrix components. Early on, Gas6 binding to Axl was shown to have a positive influence on cell-cell adhesion [25]. In fact, Axl is also known as “Ark” which stands for “adhesion-related kinase.” More recently, studies have demonstrated that the adhesion properties in which Axl is involved are both wide-ranging and context-dependent. In schwannoma, Axl cooperates with NFκB signaling to mediate cell-matrix adhesion, but in cutaneous squamous cell carcinoma, Axl mediates EMT by exerting a negative influence on cell-cell adhesion [26, 27]. Furthermore, in lung cancer cell lines Axl expression correlates with the adherence or suspension of cultures, but its expression seems to be a consequence of gaining adherent properties [28].

### Exon 11

It has been established that Axl can undergo an extracellular cleavage event in exon 11 near the transmembrane domain by an unconfirmed protease, producing a soluble fragment (Figure 1). This fragment contains both the FNIII and Ig-like domains, and is able to bind available Gas6 as a decoy receptor to effectively dampen Gas6 signaling [29, 30]. It has also been shown to bind membrane-associated Axl to inhibit signaling [31]. Soluble Axl (sAxl) has been detected in tumors, but it also may have a normal biological role in human serum where it binds to circulating Gas6 [16, 32]. Notably, application of sAxl to target Gas6/Axl signaling has been proposed as a therapeutic strategy in cancer [33, 34].

### Exons 13–20

As a Type I transmembrane receptor, Axl's enzymatic kinase domain spans exons 13–20 within the intracellular C-terminus (Figure 1). The TAM receptors share a family-specific motif in their kinase domains, about 100 amino acids downstream from the ATP active site. In Axl and Mer the amino acid sequence is KWIAIES, but in Tyro3 the isoleucines are substituted with leucines [1].

## Axl in evolution and development

*Axl* homologs have been identified in *Pan troglodytes*, *Canis lupus*, *Bos taurus*, *Mus musculus*, *Rattus norvegicus*, and *Xenopus tropicalis*, while orthologs have been identified in over 70 organisms. The TAM receptors seem to have arisen relatively recently in evolution, as they have no representation in *Drosophila melanogaster* or *Caenorhabditis elegans* [35]. The later appearance of the TAM receptors compared to other kinases such as those of the MAPK pathway supports their role in complex processes like hematopoiesis and the immune response [35].

Axl is expressed fairly late in embryogenesis compared to other RTKs. RNA *in situ* hybridization analysis has revealed the initial expression of Axl in substructures of developing tissues at day 12.5 after fertilization [36]. Whereas many RTKs are known for their essential role in embryonic development, the TAM receptor family seems to be dispensable. Triple knockout of all TAM receptors in mice lead to viable offspring, whereas even a single point mutation in developmentally required RTKs can result in embryonic lethality [37, 38]. Although the triple negative offspring live, they do develop abnormalities such as autoimmune disorders due to hyperactivation of antigen-presenting cells as well as increased B and T cell populations [39].

## Activation of Axl and downstream consequences

As a receptor tyrosine kinase, Axl is activated upon paracrine or autocrine Gas6 binding and subsequent homodimerization, causing tyrosine autophosphorylation and phosphorylation of downstream targets (Figure 2). However, the mechanism of the activation step alone has been contested in various settings. It has been thought that Axl activation and autophosphorylation can occur independently of ligand binding, such as in the experimental setting of Axl overexpression. This leads to homophilic binding of extracellular domains on opposite cells and causes cell aggregation, independent of both calcium and the Axl kinase domain [40]. In vascular smooth muscle cells (VSMCs) and lens epithelial cells ligand-independent activation of Axl occurs in response to hydrogen peroxide, where activation of Axl in the former involves reactive oxygen species (ROS) [41, 42]. In vascular endothelial cells, Axl phosphorylation in response to laminar sheer stress may occur by an association with β3 integrin [31]. Additionally, phosphorylation of Axl can occur through VEGF-A induction of a SRC family kinase member (SFK) in endothelial cells [43].

**Figure 3:**
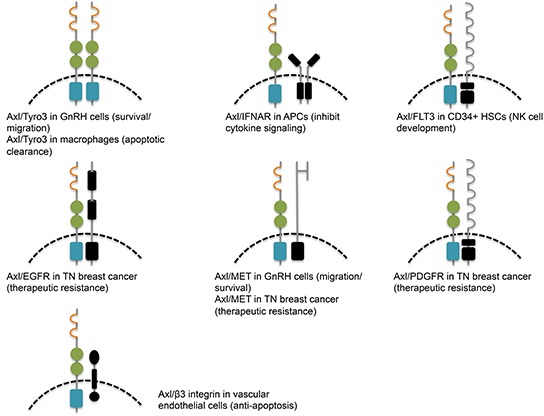
Activation of Axl by heterodimerization with plasma membrane proteins leads to cell-specific consequences

Studies have found that heterodimerization between the TAM receptors may be a mode of activation (Figure 3). Due to the differential affinity of Gas6 for the receptors, it would make sense for Axl and Tyro3 to preferentially heterodimerize. Indeed, they co-immunoprecipitate in gonadotropin-releasing hormone (GnRH) neuronal cells [44]. On the surface of macrophages, Mer phosphorylation requires Axl and Tyro3 to efficiently mediate apoptotic cell clearance, and this may be due to their heterodimerization with Mer after being stimulated with Gas6 [45]. In Rat2 fibroblast cells, Gas6 treatment induces Tyro3-dependent Axl phosphorylation, which further leads to the trans-phosphorylation of Tyro3 [46]. Heterodimerization may also occur with non-TAM receptor family members, such as the type I interferon receptor (IFNAR) and the FLT3 receptor [47, 48]. Notably, addition of FLT3 ligand leads to heterodimerization of Axl and FLT3, and phosphorylation of FLT3 is reduced after addition of the extracellular Fc portion of Axl [48]. EGFR associates with and transactivates Axl independently of Gas6 to amplify EGFR signaling in triple negative (TN) breast cancer cells [49]. Weak interaction has been proposed for Axl and MET in GnRH neuronal cells, where stimulation with their ligands Gas6 and HGF, respectively, leads to receptor-specific phosphorylation without trans-phosphorylation [50].

Aside from physical association, Axl may functionally cross-talk with other signaling pathways, leading to context-specific outcomes. In natural killer cell differentiation, Gas6/Axl signaling is involved in crosstalk with c-Kit signaling, and prevention of Gas6 binding to Axl inhibits c-Kit phosphorylation [51]. Importantly, Axl signaling synergizes with other RTKs after they are therapeutically targeted, leading to diversification of signaling and therapeutic resistance [49].

In studies to date, the signaling downstream of Axl resembles that of most RTKs. Which specific pathways are activated and at what time is context-dependent, determined by the extracellular environment, cell type, and tissue type. The initial characterization of Axl in myeloid leukemia described two PI3K consensus-binding sites in the kinase domain, similar to other RTKs [2]. Since then Axl signaling through PI3K has been firmly established in many circumstances through which it regulates cell migration, growth, angiogenesis, and apoptosis, among other processes [43, 52–55]. In 1997, Braunger et al. identified two subunits of PI3K, p85α and p85β, as well as PLCγ, GRB2, SRC, and LCK, as substrates of Axl (Table 1) [56]. These substrates all bind tyrosine 821; additionally, the p85 proteins can bind Y779 with lower affinity, and PLCγ can bind Y866 (Table 1). A yeast two-hybrid screen using the cytoplasmic domain of Axl as bait against a heart cDNA library uncovered the p55γ subunit of PI3K, SOCS-1, Nck2, RanBPM, and C1-TEN as Axl binding proteins [57]. Importantly, C1-TEN was discovered during this screen as a novel C1 domain-containing protein with homology to tensin and PTEN, and has since been implicated in cancer. By binding all of these adaptor proteins, Axl has extremely diverse signaling capabilities through the PI3K, Akt, mTOR, NFκB, and MAPK pathways. It becomes paramount, therefore, to determine the exact contribution of Axl in each tissue and disease context, and how to therapeutically manipulate it.

**Table 1:** Axl tyrosine phosphorylation and respective binding partners

Tyrosine	Potential Autophosphorylation?	Binding Partners	Reference
702	No	Grb2/Ack1	[281]
703	No	Grb2/Ack1	[281]
779	Yes	PI3K p85α/β	[56]
821	Yes/No	PLCγ, PI3K p85α/β, GRB2, SRC, LCK	[56, 282]
866	Yes	PLCγ	[56]

## Regulation of Axl

The direct regulation of Axl at the protein, translational, and transcriptional levels remains a large gap in the field. Signaling through RTKs may be dampened or shut off by a mono-ubiquitination signal, leading to endosome-mediated internalization and lysosomal degradation. This holds true for Axl signaling, whose ubiquitin ligase is c-Cbl. The Cbl family also targets EGFR, PDGFR, CSF-1R, and HGFR [58]. Binding of Gas6 to Axl promotes its downregulation through this mechanism, also common amongst other RTKs and their respective ligands [42, 59].

A similar mechanism of Axl downregulation may by imposed by the von Hippel-Lindau (VHL) protein, a ubiquitin ligase known to target hypoxia-inducible factor 1-alpha (HIF1α). Reconstitution of cells with VHL decreases Axl protein levels, but does not affect Axl mRNA levels, indicating regulation at the protein level [60]. Exclusive regulation of Axl at the protein level also occurs during chemically-induced hypoxia in prostate cancer cells. Although the exact mechanism is unknown, cobalt chloride (CoCl_2_) treatment of Gas6-stimulated cells prevents Gas6-mediated downregulation of Axl protein [61].

The use of phosphatases by the cell is a common method of reversible downregulation of RTK activity. However, there are no confirmed Axl-targeting phosphatases to date. As mentioned previously C1-TEN is an Axl binding protein with phosphatase activity, but it has not been shown to directly dephosphorylate Axl.

Post-transcriptional regulation has been shown to occur through microRNA (miRNA) binding of the 3′ UTR of Axl. So far, two Axl-targeting miRNAs have been identified, miR-34a and miR199a/b, through a bioinformatics screen using non-small cell lung cancer, breast cancer, and colorectal cancer cell lines [62]. Importantly, transfection of these miRNAs inhibits cell migration and invasion *in vitro*, and metastasis *in vivo*. Regulation by miRNAs not only has clinical significance, but it unveils another layer of diversity within Axl signaling, as expression of miRNAs is often cell/tissue specific.

**Figure 4:**
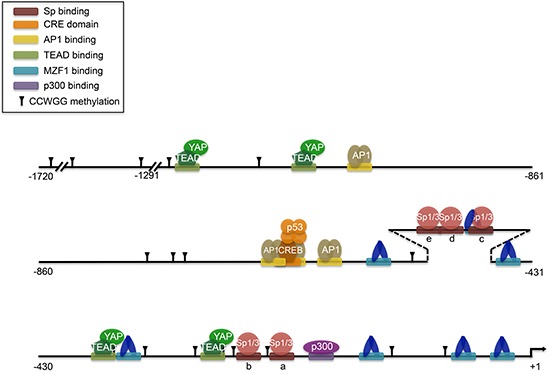
Transcription factor binding to the *Axl* promoter Adapted compilation of figures from multiple sources (Mudduluru, 2010; Mudduluru, 2011; Xu, 2011; Mudduluru, 2008; Hong, 2008; Vaughan, 2012). Putative HIF1α binding to HRE sequences is not shown. p53 interaction with CREB complex induces histone acetylation around CRE sites. YAP is a transcriptional cofactor for TEAD. Methylation of CCWGG sites are marked. CpG methylation is not shown, but occurs in 19 CpG sites within nucleotides −669 to −97. CpG methylation also occurs in Sp a, b, and c sites which prevents Sp factors from binding.

At the transcriptional level, there are multiple ways to regulate *Axl* (Figure 4). Several transcription factors have been shown to upregulate *Axl* transcription. HIF1α regulation of *Axl* was observed in a gene expression microarray using RNA from hypoxia-exposed pulmonary artery epithelial cells [63]. Although HIF1α binding to the *Axl* promoter has not been functionally validated, HIF1α binding to *Axl* was found to be enriched using ChIP-seq in human umbilical vein endothelial cells (HUVEC) under hypoxia [64]. MZF1 has been implicated in cancer development, and binding to the *Axl* promoter enhances Axl mRNA and protein expression, inducing invasion and *in vivo* metastasis in colorectal and cervical cancer [65]. AP1 is also a transcription factor that can regulate *Axl*, and is required for the overexpression of *Axl* in TKI-resistant CML cells [66, 67]. *Axl* overexpression can also occur through four TEAD-binding domains in its promoter, which requires the coactivator YAP [68]. The adenovirus type 5 early region 1A (E1A) gene exerts tumor suppressive activity by downregulating *Axl* transcription to induce apoptosis in *Axl*-expressing cells [69]. The SP zinc-finger transcription factors Sp1 and Sp3 have been shown to bind GC-rich regions in the *Axl* promoter to upregulate its transcription, while methylation of CpG sites in Sp binding regions restricts *Axl* gene expression [70]. *Axl* also has 17 CCWGG sites in its promoter, and methylation at these sites prevents Axl from responding to chemotherapy drugs [71]. Hypomethylation of the *Axl* promoter leading to upregulation is found in Karposi sarcoma cell lines [54]. The methylation status of *Axl* not only has implications for disease, but it also affects heritability, eliciting the phenomenon of genomic imprinting. A differentially methylated region (DMR) in the paternal allele of *Axl* causes silencing and preferential expression of the maternal allele [72]. Twin studies have found that DNA methylation levels of *Axl* are significantly heritable [73].

## Tissue and cell type-specific roles for Axl

Unlike its other family members, Axl is nearly ubiquitously expressed among cell types. The biologic effects of signaling through Axl, as well as consequences of Axl overactivation or downregulation, are cell/tissue type specific in health and disease (Table 2).

**Table 2:** Axl signaling and functional consequences in normal and disease contexts

Normal Cell/Tissue Type	Signaling	Consequence	Reference
HSCs	–	Hematopoietic support, inhibition of proliferation	[75]
Erythrocytes	–	Differentiation	[77]
Platelets	β3 integrin, PI3K/Akt	Aggregation	[166–168]
Megakaryocytes		Differentiation	[78]
NK cells	STAT5, c-Kit, FLT3	Differentiation	[48, 51, 84]
Macrophages	–	Apoptotic cell clearance, regulation of immune response	[86, 88, 91]
Dendritic cells	STAT1	Regulation of immune response	[47]
Chondrocytes	ERK1/2	Proliferation/differentiation	[95]
Lung (general)	MCP-1, IL-8, IFN-β, IL-13	Regulation of immune response	[6, 123, 124]
Vascular smooth muscle cells	PI3K/Akt/PKB/S6K, SHP2, PLCγ, ERK1/2	Apoptotic/injury protection, migration, survival	[157–159, 161–163, 175–177, 179, 185]
Vascular endothelial cells	c-SRC, PI3K/Akt/NFκB/Bcl2, VEGF, SHP2, β3 integrin, IFN-γ	Proliferation, apoptotic protection of quiescent cells, angiogenesis, inflammatory response	[31, 43, 53, 107, 164, 165, 181, 182, 187, 188]
Cardiac fibroblasts	ERK	Proliferation	[174]
Renal glomerular cells	PDGF	Proliferation	[191]
Renal tubular cells	–	Proliferation	[193]
Adipocytes	–	Maintenance of stemness	[201–203]
Schwann cells	ERK2	Proliferation	[221]
GnRH neurons	MEF-2, PI3K/Akt, ERK1/2	Migration, apoptotic protection	[44, 226–228]
Microglia	p38 MAPK, NFκB	Apoptotic clearance, inhibition of cytokine signaling	[224, 231–233]
Cerebral endothelial cells	Akt	Apoptotic protection	[234]
Oligodendrocytes	PI3K/Akt	Apoptotic protection	[235, 236]
Lens epithelial cells	PI3K/Akt	Proliferation, survival, development	[245, 246]
Retinal cells	–	Clearance of apoptotic cells	[90, 247, 248]
Hepatic oval cells	–	Apoptotic protection	[266]
Hepatic stellate cells	PI3K/Akt, NFκB	Apoptotic protection	[269]
Liver (general)	SOCS1	Regulation of immune response	[5, 271]
Disease Cell/Tissue Type	Downstream Signaling	Consequence	Reference
AML	FLT3, Akt, MAPK, IL-10, M-CSF	Proliferation, maintenance of stemness, therapeutic resistance	[33, 79, 80]
CML	–	Therapeutic resistance	[66]
B cell-derived microvesicles in CLL	PI3K, SRC, PLCγ2, Akt	Apoptotic protection	[82, 83]
Osteosarcoma	Akt, MMP-9	Apoptotic protection, invasion/migration, proliferation	[96–99]
Prostate cancer	PI3K/Akt/NFκB, MAPK	Proliferation, invasion/migration, dormancy, therapeutic resistance	[100–104]
Breast cancer	NFκB, c-MET, PDGFR, EGFR, MMP-9, SOCS	EMT/migration, proliferation, apoptotic protection, therapeutic resistance	[49, 69, 108, 109, 112–117, 120–122]
Mesothelioma	PI3K/Akt/mTOR, MAPK	Migration/invasion, proliferation	[126, 127]
NSCLC	ERK, PI3K/Akt/Rac1, NFκB, EGFR	Regulation of immune response, proliferation, EMT/migration, therapeutic resistance	[62, 129–132, 136–138, 140–142, 146, 147, 149]
Renal cell carcinoma	–	Proliferation, angiogenesis	[60, 196–200]
Melanoma	STAT3, RAF/MEK, NFκB	Migration/invasion, apoptotic protection, therapeutic resistance	[210, 212–215, 217]
Squamous cell carcinoma	Akt/Blc2, Wnt/TGFβ, NFκB	Migration/invasion, apoptotic protection, therapeutic resistance, disruption of cell-cell adhesion	[27, 93, 220]
Schwannoma	FAK/Src/NFκB	Proliferation, cell-matrix adhesion	[26]
Astrocytoma	PI3K/Akt, ERK1/2	Therapeutic resistance, apoptotic protection	[230]
Glioma	–	Migration/invasion, survival, maintenance of stemness, angiogenesis	[158, 237–241]
Ocular melanoma	Cyr61	Apoptotic protection, proliferation	[251]
Retinoblastoma	–	Proliferation	[252]
Thyroid carcinoma cells	–	Apoptotic protection, proliferation, invasion, angiogenesis	[253–256]
Colon carcinoma	STAT3, SFK, PI3K/Akt	Proliferation, invasion, therapeutic resistance	[65, 258–263]
Hepatocellular carcinoma	Cyr61, ERK, PI3K/Akt	Migration	[274, 275, 277]

### Hematopoiesis

**Figure 5:**
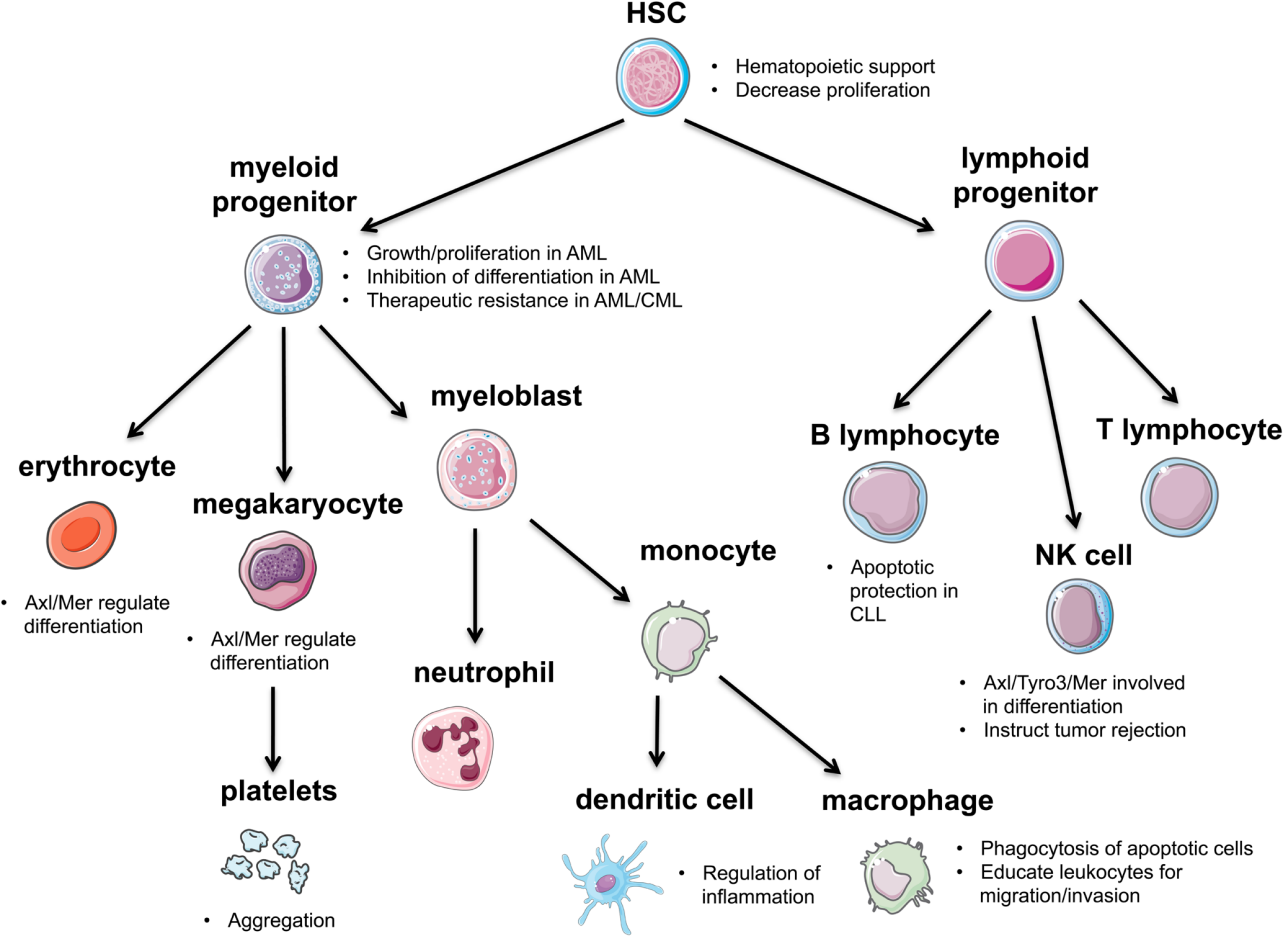
Representation of Axl in the blood-forming lineages Axl contributes to the maintenance of HSCs and helps regulate differentiation of various HSC lineages. Axl plays a major role in the immune response by regulating inflammation and helping to clear apoptotic cells.

The initial discovery of *Axl* in a screen for CML transformants alludes to its involvement in the blood-forming lineages (Figure 5). Since then, an important role for Axl has been established in maintaining normal homeostasis of hematopoiesis, and it is most prominent in the CD34^+^ early myeloid lineage of hematopoietic cells [74].

Most likely through Axl signaling, Gas6 production by bone marrow stromal cells supports hematopoiesis in culture [75]. Support of hematopoiesis was defined in this study as the ability to produce myeloid colony-forming cells for months [76]. Since the soluble form of Gas6 is not sufficient for hematopoietic support, the mechanism is proposed to be through Gas6-mediated chemotaxis of Axl-expressing progenitor cells [75]. Both Axl and Mer cooperate to regulate the differentiation of cells in the erythroid lineage, where *Axl*^−/−^ *Mer*^−/−^ mice are unable to produce differentiated erythroid progenitors [77]. These mice also display impaired megakaryocytopoiesis indicated by prolonged time to clot after transection of the tail tip compared to normal or other combinations of double knockout mice [78].

Axl represents a prognostic biomarker in diseases of the myeloid lineage such as AML and CML, and may be a therapeutic target. *Axl* is upregulated in AML patients and correlates with a decrease in both progression-free and overall survival [79]. In FLT3/ITD-driven AML, Axl positively regulates constitutive FLT3, leading to cell growth, proliferation, and inhibition of myeloid differentiation [33]. Another study found that Axl can be therapeutically targeted in AML independent of the FLT3 mutational status [80]. Furthermore, this therapeutic inhibition of Axl also inhibits the Akt and MAPK pathways, implying a mechanism through which *Axl* upregulation promotes growth and proliferation in AML cells [80]. A mode of chemoresistance in AML cells has been proposed to be through their instruction of bone marrow stromal cells to upregulate Gas6 through IL-10 and M-CSF [80]. In CML, resistance to the TKI imatinib is also correlated with high levels of Axl [66]. The reoccurrence of the participation of Axl in therapeutic resistance may also indicate its role as more of a “passenger” than a “driver” of disease. In fact, a study to determine if Axl participates in the onset or the progression of CML became a study of noninsulin-dependent diabetes mellitus (NIDDM), when ectopic overexpression of Axl in the myeloid lineage of transgenic mice induced a NIDDM phenotype rather than causing hematopoietic malignancies [81]. Although it is more frequently expressed in myeloid cells, inhibition of Axl in B cell-derived microvesicles has been shown to increase apoptosis in CLL B cells [82]. However because of the cooperative nature of Axl, these apoptotic effects may be mediated by combination signaling with the other upregulated kinases in CLL such as PI3K, c-Src, and PLCγ2 [82]. Additionally, B cell-derived microvesicles circulating in CLL plasma can deliver constitutively phosphorylated Axl to BMSCs to enhance the tumor microenvironment and recruit additional tyrosine kinases, and not surprisingly, this phosphorylation status correlates with clinical prognosis [82, 83]. Axl expression also has implications in other cells of the lymphoid lineage, such as natural killer (NK) cells. In combination with Tyro3 and Mer, Axl signaling is essential for the differentiation of NK cells, where it regulates the IL-15, c-Kit, and FLT3 pathways [48, 51, 84]. Interestingly, NK cells can be instructed to reject metastatic tumors through the inhibition of Cbl-b, an E3 ubiquitin ligase for all three TAM receptors [85].

### Myeloid lineage

Macrophages are differentiated cells of the myeloid lineage, and of the TAM receptors, mutated *Mer* seems to have the most pronounced effect on macrophage function [86]. However, given that Axl is preferentially expressed in macrophages, monocytes, and dendritic cells compared to cells of the lymphoid lineage, its role in the innate immune response cannot be overlooked. Macrophages are an important component of the immune system, in that they ingest foreign material and can act as antigen-presenting cells. Triple knockouts of the TAM receptors in mice remain embryonically viable, but develop autoimmune diseases around 4 weeks postnatal [86]. Specifically, the inability of macrophages to phagocytose and clear apoptotic cells reflects the normal role of the TAM receptors in this process, although it is more attributed to the Mer receptor [86]. The Gla domains of both Gas6 and protein S are able to bind phosphatidylserine (PS) and acidic phospholipids which become expressed on the outer leaflet of the plasma membrane during apoptosis [87]. It is proposed that the ligands are still available for TAM receptor binding, which under normal circumstances leads to mobilization of apoptotic cells to macrophages which have upregulated TAM receptors as a response to the initial immune stimulus [86]. This mechanism becomes hijacked in infertile men in which increased levels of estradiol stimulate Leydig cells to produce more Gas6 and elevate levels of PS on their surfaces, independent of the apoptotic status of the cells [88]. In this setting Axl acts as the primary receptor for bridging Gas6 with testicular macrophages. Additional ligands for the TAM receptors, tubby and Tulp1, have been more recently discovered for their role as macrophage phagocytosis ligands, acting in a similar manner to Gas6 and protein S in their ability to bridge macrophages with apoptotic cells [89, 90]. Tubby is specific for Mer, but Tulp1 can interact with any of the TAM receptors. Additionally, Axl has a normal role in regulating innate immunity by limiting cytokine-mediated inflammation. This is in part due to the activity of the JAK/STAT pathway, whereby TLR-driven cytokine activation leads to STAT/IFNAR-dependent transcription of Axl in dendritic cells [47]. Upregulation of Axl by Type I interferon (IFN) is required for IFN downregulation of TNF-α production, hence the anti-inflammatory role of Axl in the innate immune response [47]. This extends positively as a protective role in colitis and colorectal adenomas, the risks of which are both dramatically increased with chronic inflammation. Axl and Mer cooperation in lamina propria macrophages helps to regulate the inflammatory immune response, as *Axl*^−/−^*Mer*^−/−^ mice have a significant increase in proinflammatory mediators [91].

Macrophages can also interact with tumor cells to promote malignancy. Tumor-associated macrophages express and secrete high levels of Gas6 in the tumor stroma, possibly to help educate infiltrating leukocytes to increase their production of Gas6, and this cooperative Gas6 increase in the bone marrow niche promotes tumor growth and metastasis of cells expressing high Axl [92]. In oral squamous cell carcinoma (OSCC) cells, expression of Axl is increased during coculture with tumor-associated macrophages having abundant levels of Gas6 [93]. This stimulates Axl signaling through NFκB to promote malignancy. Taken together, this suggests that tumor cells can exploit Axl by increasing the availability of its ligand through macrophages in order to create a supportive environment for tumor growth and survival. Again, this is consistent with a more secondary role for Axl, where it becomes a factor in cancer progression and maintenance, rather than initiation.

### Bone

As alluded to previously, Axl/Gas6 signaling is an important part of the interaction between bone marrow derived hematopoietic stem cells and bone marrow stromal cells. Because of the heterogeneous nature of the bone marrow, the autocrine/paracrine secretion of Gas6 becomes an important aspect of Axl signaling and communication between different cell types. This is a normal component of the bone marrow microenvironment, but is also exploited to support cancer development and maintenance. *Axl* expression was observed to be greater than 800-fold higher in bone marrow mesenchymal stromal cells (BMMSCs) compared to bone marrow-derived hematopoietic stem cells, and *Gas6* expression is similarly increased in BMMSCs around 380-fold [94]. The maturation of chondrocytes is another alternative step in the differentiation of mesenchymal stem cells in the bone marrow, and this process correlates with the expression of Axl and Mer [94, 95]. A study in bovine tissue found differential regulation of chrondrocyte differentiation by Gas6, and that this was due to opposite expression of Axl and Mer at different times [95]. Specifically, Gas6/Axl signaling in the earlier phase was suppressive of differentiation, whereas in the later phase Gas6/Mer signaling was supportive of differentiation.

Although a role for Axl in osteocyte differentiation from mesenchymal stem cells has not been proposed, its expression in osteoblasts is significant in cancer. Not unlike other cancers, osteosarcoma cells show increased levels of activated Axl which are correlated with clinical prognosis [96]. In this setting, Axl protects tumor cells from apoptosis and promotes their invasion and migration, potentially contributing to lung metastasis. Phosphorylated Axl may mediate these effects through Akt signaling and upregulation of matrix metalloproteinase 9 (MMP-9). In the same manner, another study showed that knockdown of *Axl* increased apoptosis and decreased proliferation, also mediated by downregulation of the Akt pathway [97]. A recent study unraveled additional RTKs involved in the metastatic potential of osteosarcoma, and after lengthy metastatic-dependent validations *Axl* came out as a top hit in a metastatic osteosarcoma cell line [98]. *Axl* also emerged from another screen for differentially expressed genes in high versus low metastatic osteosarcoma sublines [99].

In a similar manner to the exploitation of macrophages by cancer cells to create a supportive tumor niche, osteoblasts can support disseminated prostate tumor cells (DTCs) that metastasize to the bone marrow. There, osteoblasts physically bind DTCs through annexin2, and this causes upregulation of *Axl* transcription and display on the DTC surface [100]. This allows for sufficient, localized binding of Gas6, which is secreted by the osteoblasts. However, unlike the pro-proliferative properties of Axl in other cancers, Gas6/Axl signaling in this cellular context leads to tumor cell dormancy and evasion of therapy.

### Prostate

The contribution of Axl to processes in the normal prostate has not been studied, but in prostate cancer, Axl has clinically significant implications. The first account of Axl in prostate cancer was its elevated expression in a metastatic prostate carcinoma cell line, DU145, compared to normal prostate cells and another prostate carcinoma cell line, PC3 [101]. This has since been confirmed by other studies. On the other hand, the mRNA levels of *Gas6* are unchanged between normal and prostate cancer tissue [102]. Notably, Axl activation also correlates with the androgen-insensitive cell lines PC3, DU145, and CL1 [102]. As well as possessing a putative role in metastasis, Axl has been shown to increase proliferation of prostate cancer cells. Both PC3 and DU145 cells respond to Gas6 by increasing their proliferation, but this effect is more predominant in DU145 cells in correlation with elevated levels of Axl [103]. This mitogenic signaling is through a combination of the PI3K/Akt and MAPK pathways, and is therefore complicated by the common occurrence of PTEN deletions in prostate carcinomas and the PC3 cell line [103]. Further investigation into the mitogenic signaling downstream effector of Axl/Akt activation in prostate cancer uncovers NFκB, which also induces the secretion of IL-6 to activate IL-6/STAT3 signaling [102]. Silencing *Axl* in PC3 cells inhibits proliferation, invasion, and migration; implantation of *Axl*-silenced PC3 cells into mice displays reduced cell growth compared to mice implanted with untransfected PC3 cells [102]. Unlike in other cancer types, Gas6/Axl signaling is unable to protect prostate cancer cell lines from serum starvation-induced apoptosis [103].

Prostate cancer preferentially metastasizes to the bone, leading to devastating morbidity and mortality. In mouse models of human prostate cancer, Axl expression is increased in DTCs compared to the primary tumor, where Tyro3 expression prevails [104]. Proliferation appears to correlate with increased Tyro3 levels, whereas decreased Ki67 staining correlates with increased Axl levels, implying dormancy of DTCs [104]. It is thought that the mechanisms governing HSC quiescence and hematopoiesis are transferrable to DTC dormancy, and this supports the involvement of Gas6/Axl signaling [75, 104]. As mentioned earlier, Gas6 is secreted by osteoblasts in the bone marrow, which increases *Axl* expression in DTCs upon binding to annexin A2 [100]. The tendency of prostate metastases to be osteoblastic rather than osteolytic, further builds a more suitable cancer niche to facilitate DTC dormancy through a Gas6/Axl axis. The stability of Gas6/Axl signaling is thought to be mediated by hypoxia, where the hypoxia-mimicking agent, CoCl_2_, prevents the c-Cbl-mediated downregulation of Axl [61]. This downregulation of Axl is exclusively at the protein level, as *Axl* mRNA levels are unchanged. It is undetermined whether the chemically-induced stabilization of HIF1α is an accurate reproduction of the hypoxic environment in terms of Axl stabilization. This will be an important feature to determine, as the bone marrow exhibits reduced oxygen levels. Dormancy through Gas6/Axl signaling may be involved in mediating protection against chemotherapy, as treatment of PC3 cells *in vitro* with Gas6 decreased chemotherapy-mediated apoptosis [100]. Axl has been implicated as a prognostic and imaging marker in some cancers, including prostate cancer. A protein array detecting antigens from serum of immunized mice identified Axl as a strong candidate [105, 106].

The dichotomous effects of Axl in proliferation in prostate cancer models suggests that its role most likely depends on the exact cellular context. Studies that observe pro-proliferative effects by Axl seem to focus on the primary tumor, whereas cell dormancy mediated by Axl occurs in the bone marrow. It could also be that Axl is merely *associated* with DTC dormancy in the bone marrow, where it acts as a pro-survival factor to keep cells from undergoing apoptosis until they receive further signals to reawaken. This notion has been implicated in the quiescent endothelial cells of the vessel wall [107]. Additionally, proliferation assays *in vitro* of PC3 cells with Gas6 yield opposite results in different studies [103, 104]. This may simply be due to discrepancies of the assays, or more significantly, to the sensitivity of Axl to the surrounding environment. Regardless, it will be important to determine the regulation of Axl and Gas6 in these contexts in order to fully understand the contribution of Axl signaling to the progression of prostate cancer. Axl may represent a therapeutic target in preventing metastasis.

### Breast

Axl is expressed in the normal mammary gland, but many studies have detected its overexpression in aggressive tumors, cell models of breast cancer, and metastatic tumors, and thus, it may independently predict reduced patient survival [108–112]. This has led to the emergence of multiple Axl inhibitors for research and therapeutic use across many cancers. It is worth noting that Axl is expressed exclusively in breast epithelial cells, and not in the surrounding fibroblasts or adipose tissue [109]. Although Axl is upregulated in TN breast cancer cell lines, this correlation does not carry over to patient samples [113]. Instead, membranous expression of Axl is associated with lymphovascular invasion, implying a role in migration and metastasis [113]. Interestingly, increased expression of Axl in metastasized breast cancer has been shown to be an effector of metastasis, where it maintains invasiveness rather than functioning as a driver [108]. This is attributed to the induction of *Axl* by EMT-inducing transcription factors, and a follow-up study implicated vimentin as an intermediary between the two [108, 114]. Microarray analysis in breast epithelial cells showed decreased expression of *Axl* after knockdown of *vimentin*, and that *Axl* and *vimentin* correlate positively in patient samples [114]. Functional studies further define Axl as an important regulator of migration, and some even place Axl upstream as an inducer of EMT [114, 115]. In inflammatory breast cancer cells, TIG1 stabilizes Axl by inhibiting its proteasome-dependent degradation; this reduces proliferation, migration, and invasion of the cells through NFκB and MMP-9 activation [116]. Regulation of Axl to promote invasion and metastasis can also occur via glycosylation, as evidenced by inhibition of ST6GalNAcII resulting in decreased Axl expression and invasive ability in malignant tumor cells [112].

Axl is necessary for the tumorigenesis of breast cancer cells *in vivo*, and this extends to maintaining tumor growth despite apoptotic signals induced by nutrient deprivation [69, 117]. Overexpression of Axl protects breast cancer cells from serum starvation-induced apoptosis, as it does in osteosarcoma cells, mentioned previously [69, 109, 117]. Negative regulation of *Axl* by the early region 1A (E1A) gene mediates the pro-apoptotic, tumor suppressive properties of E1A in breast cancer, whereas estrogen induction of *Axl* protects breast cancer cells from apoptosis [69, 109]. Axl and the estrogen receptor (ER) have high expression correlation in a subset of breast cancer, and treatment of ER+ cells with an ER antagonist or depriving them of estrogen decreases Axl expression [109, 118].

The study of Axl in breast cancer has led to the development of novel therapeutics, as well as a role in therapeutic resistance to other targeted therapies. Initial Axl-targeting strategies have employed anti-Axl polyclonal antibodies, and small molecule inhibitors which target multiple TKRs [117]. Since then, anti-Axl monoclonal antibodies have been developed, which have further validated the role of Axl in tumor growth and metastasis in breast cancer xenograft tumors [119]. Touching on the negative role of the TAM receptors in innate immunity, implications for immunotherapy in breast cancer have arose based on the observation that therapeutic-resistant cancer stem cells have increased signaling through cooperation of SOCS and the TAM receptors, making them more susceptible to oncolytic adenovirus [120]. The use of bi-specific inhibitors was also proposed when it was discovered that Axl phosphorylates c-MET in response to Gas6 in TN breast cancer cells [121]. MP470 is a multikinase inhibitor which targets Axl, mutant *c-KIT*, and PDGFRα, and reverses EMT in breast cancer stem cells through the NFκB pathway [115]. On the other hand, a selective Axl inhibitor, R428, is still able to inhibit metastasis and angiogenesis [122].

For many women, breast cancer therapy targets the HER2 receptor, but this treatment almost always eventually fails secondary to resistance by mechanisms which are currently being explored. Axl overexpression appears to be a contributor to resistance. Blocking Axl with the multikinase inhibitor GSK1363089 restores sensitivity to HER2/ER positive cells originally treated with lapatinib and trastuzumab [118]. Furthermore, Axl was identified from a database as being predictive of a lack of response to therapies targeting the ERBB receptor family. It was shown to be physically associated with, and transactivated by EGFR, leading to diversification beyond EGFR signaling alone [49]. Notably, Axl also associated with MET and PDGFR.

### Lung

The regulation of the innate immune system by Axl has implications in the lung. Activation of Axl decreases inflammation in a cell model of LPS-induced acute lung injury through the inhibition of cytokine signaling [6]. In respiratory viruses, an anti-Axl monoclonal antibody is able to extinguish many consequences of infection by boosting the antiviral immune response with type I IFN, as well as inhibiting allergic inflammatory responses [123]. Further support for targeting Axl in respiratory diseases comes from a subsequent study in which Gas6 plasma levels are elevated by M2 macrophages in clinical asthma, driving T cell activation through Axl expression on dendritic cells [124].

Pleural mesothelioma is a cancer that is highly dependent on RTK signaling for proliferation, and is often chemotherapy-resistant [125]. Axl is among the RTKs involved, and its expression is found to be higher than in other cancers in which it plays a role [126]. Inhibition of Axl in mesothelioma cell lines inhibits migration and invasion, but the major function of Axl in mesothelioma is to promote proliferation [126]. Axl induces proliferation through a PI3K/Akt/mTOR axis, and inhibition of Axl leads to G1 growth arrest [126, 127]. Overexpression of Axl alone is able to predict patient survival, but selective RTK inhibition in mesothelioma has not been effective, as in the case of EGFR inhibitors [125, 128]. Therefore, targeting an array of RTKs might be a more suitable approach to take in treating mesothelioma patients [125].

One of the earliest accounts of Axl in lung cancer was due to its expression correlation in adherent cultures of lung cancer versus suspension cultures, owing to the structural adhesion features in its extracellular domain [28]. However, Axl expression seems to be a consequence of inducing adhesion in suspension cultures, which also correlates with the type of lung cancer. Non-small cell lung cancer (NSCLC) grows as adherent cultures, whereas small cell lung cancer (SCLC) grows in suspension. Since then, Axl expression has been shown to correlate with many features of NSCLC [129–132]. Both protein and mRNA levels of Axl are associated with poor prognosis and pathological features of lung adenocarcinoma [131]. This holds true for Gas6 protein levels, whereas high Gas6 mRNA levels are actually related to better clinical outcome for patients [131]. Gas6 secretion by exogenous sources in the surrounding tumor environment may explain this inconsistency [131]. Furthermore, activated Axl is detected in the majority of lung adenocarcinoma cases, and correlates with increased tumor size [130]. In fact, recent *in vivo* imaging of Axl using an anti-Axl antibody in lung cancer xenografts has demonstrated to be of use in diagnosis, prognosis, and tumor monitoring [133].

Axl-mediated tumor growth is predicted to be through ERK, as an anti-Axl mAb inhibits its activation and decreases proliferation *in vitro* [130]. Axl may be positively regulated by YES-associated protein 1 (YAP1); knockdown of YAP decreases both Axl and PCNA expression, and inhibits proliferation of lung adenocarcinoma cells [129]. Different mutated forms of *p53* frequently drive lung cancer, and functional analysis has demonstrated that *Axl* is a transcriptional target of both WT and mutant p53 [134]. Thus, *Axl* is induced by a driver of tumorigenicity, presenting a notion that drivers of cancer must have the ability to be mutated, whereas the lack of Axl activating mutations may place it in more of a passenger position, being recruited for cancer maintenance. Aside from transcriptional and translational regulation, a potential method of Axl activation has been found by transcriptome sequencing of primary lung adenocarcinomas, unveiling a novel fusion gene of *Axl* and *MBIP* [135]. The fusion gene retains the kinase domains and dimerization units, necessary for activation. Further investigation into the causes and consequences of this fusion event is warranted.

Axl is also involved in EMT and migration of lung cancer. Metastases of the lung usually end up in the lymph nodes, and Axl expression is seen to correlate with this status [132, 136]. Transfection of miR-34a and miR-199a, which target *Axl*, inhibits invasion in metastatic lung cancer cells and *in vivo* metastasis [62]. Migration may also occur through ROS activation of Axl with subsequent activation of PI3K/Akt and Rac1, and this would represent an adaptive characteristic of the tumor cell to oxidative stress [137]. Overexpression of *Axl* in cells induces filopodia formation and EMT-like morphology, and their invasive potential is dependent on the first Ig domain on the N-terminus and on the kinase domain, but not on the two FN3 domains [138]. Furthermore, Axl signaling through NFκB might be part of this mechanism, as treatment with a NFκB inhibitor diminished the Axl-mediated invasiveness [138].

The participation of Axl in EMT is implicated in mechanisms of chemotherapy and TKI resistance. Since significant time has passed since the development of TKIs such as erlotinib and gefitinib, the overwhelming majority of Axl studies in lung cancer attempt to elucidate mechanisms of acquired resistance. More recently, Axl has been added to the list of common contributors of TKI resistance in lung cancer such as secondary mutations in EGFR, and overactivation of other genes like MET, HGF, and IGF1-R [139]. Patients with developed resistance to erlotinib due to mutations in *EGFR* also have increased levels of Axl [136, 140]. In these patients, Axl also promotes EMT, and inhibition of Axl restores erlotinib sensitivity in tumor models [140]. Axl can also act as a binding partner with EGFR upon HGF treatment in EGFR-mediated TKI-insensitive models, potentially contributing to a mode of resistance [141]. Chemotherapy resistance can influence acquired resistance to TKIs, and Axl has also been shown to increase cell motility in this setting [142]. Whether Axl upregulation is an overall inducer or a consequence of EMT is not clear, and may depend on the particular experimental or physiological setting. Studies of EMT in TKI resistance in lung cancer typically have implicated Axl as a marker of EMT, joining the rank of snail, twist, vimentin, and N-cadherin, to name a few [143–145]. EMT as a driver of erlotinib resistance has been proposed, where Axl is part of an EMT signature in resistant mesenchymal cells, which consequently have greater sensitivity to the Axl inhibitor, SGI-7079 [146]. Expression of Axl-altered miRNAs can induce resistance as well as EMT morphology and functional characteristics in gefinitib-sensitive cell lines, indicating a role for Axl as an EMT driver [147]. However, one study found that knockdown of *Axl* in generated erlotinib-resistant cells did not restore their sensitivity [148]. Protection from apoptosis as a mechanism of therapeutic resistance includes Axl as well – knockdown of *Axl* leads to NSCLC sensitivity by increasing apoptosis [149].

The role of Axl in lung cancer and in resistance to current therapies have sparked the development of Axl inhibitors for research and clinical use [150]. A high-throughput, high-content screen based off of Gas6-induced phosphorylation of Akt in a NSCLC cell line was recently developed as a tool for identifying potential new therapeutics [151]. Axl was recently identified as a target of apigenin, a natural product of plants with implications in chemoprevention [152, 153]. Another natural compound, epigallocatechin gallate (EGCG), induces cytotoxicity in lung cancer cells whether treated with or without cisplatin, by suppressing both Axl and Tyro3 [154]. Similarly, Met and Axl can both be targeted by NPS-1034 in cells with acquired resistance to TKIs, leading to cell death [155]. A novel approach to Axl inhibition is the development of aptamer-miRNA conjugates, whereby targeting *Axl* reduces growth of tumor xenografts [156].

### Vascular smooth muscle/endothelial cells

Axl is highly expressed in vascular smooth muscle cells (VSMCs) and serves a protective role during vascular injury [157–159]. As in other cellular contexts, Axl exerts anti-apoptotic effects to mediate a variety of processes. Constitutive phosphorylation of Axl is observed in growth-arrested pulmonary artery endothelial cells, and addition of Gas6 further increases phosphorylation of Axl and leads to greater cell viability [107]. Inorganic phosphate (Pi) induces VSMC calcification through apoptosis, initially found to be through downregulation of Gas6/Axl interaction which can be restored by statins [160–162]. In the absence of Pi, normal anti-apoptotic effects are specifically due to Gas6/Axl activation of the PI3K/Akt pathway, leading to activation of NFκB and the anti-apoptotic member Bcl2 [163, 164]. Acidification as a result of hypercarbia also inhibits cellular apoptosis through Gas6/Axl activation [165]. Mild acidification can be a result of laminar sheer stress due to tangential blood flow against vascular endothelial cells, leading to an array of signaling cascades and anti-apoptotic effects mediated by Axl [31]. Axl is upregulated in cells undergoing laminar stress compared to those in static flow, and its phosphorylation is independent of Gas6 [31]. Instead, Axl physically associates with β3 integrin, promoting its own phosphorylation and anti-apoptotic effects [31]. Axl and its other family members can also stimulate the phosphorylation of β3 integrin to promote platelet aggregation, potentially through their activation of PI3K/Akt, known to play a role in this process [166–168].

Restoring Axl function may be a therapeutic strategy in patients with disease linked to calcified blood vessels, such as atherosclerosis, diabetes, and kidney disease [169]. Axl and Gas6 upregulation may contribute to the observed reduction in atherosclerotic events within the left internal mammary artery, used for coronary artery bypass grafting, compared to in the aorta [170]. Advanced atherosclerotic plaques show decreased expression of Axl, whereas the expression of Mer and protein S is increased, consistent with the abundance of protein S known to be in the plasma [171]. Another study found that plasma Gas6 levels may be used as a biomarker in atherosclerotic disease, due to its correlation with high Gas6 and Axl expression in the aorta of CABG patients [172].

Response to vascular injury requires efficient migration and proliferation of cells, and this is mediated by signaling of growth factors through RTKs. Upregulation and secretion of Gas6, and subsequent activation of Axl is one aspect of vascular injury response. Both Axl and sAxl are upregulated in heart failure patients, and sAxl may be of use as a diagnostic marker [173]. Cardiac fibroblasts respond to Gas6 and increase their proliferation through Axl-activated ERK [174]. *Axl* can be increased by thrombin or angiotensin II (Ang II), and activation by Gas6 leads to cell proliferation at the site of injury [175, 176]. Furthermore, the time over which Gas6/Axl signaling increases after injury parallels the timeline of the neointima formation [157]. Axl increases proliferation of cells by inhibiting apoptosis, and again, this is through activation of the PI3K/Akt pathway rather than through ERK1/2 [177]. In addition to mediating apoptosis at sites of vascular trauma, Axl signaling can regulate immune heterogeneity of vascular cells, the expression of cytokines and chemokines, and remodeling of the ECM [178]. Another important feature of vascular remodeling is the ability of cells to migrate, and just as Axl mediates EMT and migration in cancer models, it is able to increase migration in VSMCs by interacting with the myosin heavy chain (MHC)-IIB in response to Gas6-stimulated ROS production [179]. Oxidative stress plays a large role in vascular disease, as ROS is an important signaling molecule. In the setting of vascular injury, activation of Axl by ROS is partially ligand-independent, where inhibition of Gas6 somewhat decreases Axl phosphorylation [41]. ROS-activated Axl contributes to vascular pathology, making Axl an attractive therapeutic target [180].

Vascular remodeling in response to hypertension also uses Axl signaling to protect against apoptosis, but this contributes to endothelial dysfunction [181]. Increased vascular apoptosis in mice lacking *Axl* display lower systolic blood pressure [181]. Furthermore, hematopoietic expression of Axl is responsible for the initiation of salt hypertension due, in part, to the upregulation of IFN-γ [182]. Thus, in this setting, Axl *promotes* the inflammatory response, unlike its normal anti-inflammatory role in the immune system. During pregnancy, severe preeclampsia correlates with elevated levels of plasma sAxl [183]. This form of Axl is complexed with Gas6, making it unavailable for signaling. Endothelial damage is supposedly a hallmark of preeclampsia, but the implications of reduced Axl signaling in this context are yet to be explained. Hypertension can also be a result of diabetes, in which VSMC signaling is altered by changes in glucose levels [184]. Glucose affects Axl signaling by altering its interactions with its binding partners – in low glucose, Axl associates with PI3K, but increased glucose leads to interaction with protein tyrosine phosphatase SHP-2 [185]. Consequently, Axl/PI3K interaction leads to increased cell survival, and Axl/SHP-2 interaction leads to increased migration through activation of ERK1/2 [185]. Another study found that high glucose is inversely correlated with plasma Gas6 levels, leading to decreased Axl signaling through Akt and increased adhesion in human microvascular endothelial cells [186].

Angiogenesis is a key feature of tumor growth, whereby vascular endothelial cells gain the ability to proliferate off of and extend existing vessels. Axl was first implicated in the process of angiogenesis in a search for RTKs expressed in the rheumatoid synovium of rheumatoid arthritis patients [187]. Gas6 was shown to protect human umbilical vein endothelial cells (HUVECs) from TNFα-mediated apoptosis [187]. Functional interaction with VEGF activates SFKs to mediate ligand-independent Axl activation and subsequent PI3K/Akt signaling [43]. However, Gas6 has been implicated as a negative regulator of angiogenesis, whereby stimulation of Axl in vascular endothelial cells results in the reversal of ligand-mediated VEGF activation by recruiting the tyrosine phosphatase SHP-2 [188].

In cancer models, simultaneous inhibition of Axl and VEGF effectively impairs tube formation, suggesting a potential method of intervention to prevent tumor growth and metastasis [119, 189]. Given that hypoxia drives angiogenesis within tumors, it is interesting to consider the role of Axl in response to hypoxia. As mentioned earlier, HIF1α has been shown to bind *Axl* by ChIP analysis, and Axl signaling is stabilized in prostate cancer cells after treatment with CoCl_2_, a stabilizer of HIF1α. Thus, within a tumor, the stabilization or upregulation of Axl by the hypoxic environment could also help to further promote angiogenesis.

Post-transcriptional upregulation of Gas6 is found to occur after lactate addition to HUVECs, and this engages Axl to promote PI3K/Akt signaling in angiogenesis [53]. An early study proposed Gas6 to be a chemoattractant for the migration of primary vascular endothelial cells, but without possessing significant mitogenic potential [190]. However, the first demonstration of VEGFR2-Axl crosstalk found that Gas6-activated Axl was antagonizing for vascularization, and that Gas6 inhibited chemotaxis of endothelial cells [188].

### Kidney

Axl is involved in various diseases of the kidney. Control of proliferation by Axl has been observed in glomerulonephritis, where treatment of mice with a low dose of warfarin inhibits glomerular proliferation [191]. Axl is upregulated in acute tubular necrosis associated with chronic rejection following renal transplantation, and this coincides with Gas6 levels [192]. Furthermore, albuminuria due to podocyte loss results in proliferation of the tubule cells as an adaptive response, possibly associated with the observed increase in Axl phosphorylation [193]. Axl is also localized to the tubular segments of the medulla after treatment of mice with an angiotensin-converting enzyme (ACE) inhibitor, used to prevent renal tubule atrophy, and an inhibitor of nitric oxide synthesis [194]. However, the upregulation of Axl in this situation is an unexpected result, as findings from a previous study observed upregulation of Axl and Gas6 by Ang II and subsequent downregulation after inhibition of NADPH-oxidase [195]. These findings may heavily depend on the type of renal injury, but implicate Gas6/Axl signaling as an important aspect of renal disease.

The oncogenic role of Axl extends to renal cell carcinoma (RCC), where its expression is increased compared to in the normal kidney [196, 197]. Specifically, patients with low *Axl* and high Gas6 mRNA levels in the tumor have better prognosis than those without [198]. Better prognosis is also observed in patients with both low sAxl and Gas6 in their serum, but it is unknown whether these levels are affected by the output of surrounding cells [198]. Clear cell renal cell carcinoma (ccRCC) is often found to display genetic alteration of VHL, increasing angiogenic potential by stabilizing HIF1α and HIF2α, and increasing VEGF expression [199]. In a ccRCC cell line, expression of functional VHL decreases Axl protein levels, but *Axl* mRNA levels are unchanged [60]. Since VHL is a ubiquitin ligase, it may target Axl for protein degradation. The biology of Gas6/Axl signaling in ccRCC is complicated by the fact that it has been shown to inhibit VEGFR-dependent angiogenesis in vascular endothelial cells, but again, this may depend on cell type [60, 188]. Another study found that Axl expression is dependent on VHL in RCC, and that higher expression in endothelial cells correlates with better clinical outcome, consistent with Axl being an antagonist of angiogenesis in epithelial cells [200].

### Adipocytes

A general role of the TAM family has more significant consequences in adipose tissue compared to the somewhat controversial role of Axl alone. The first characterization of Gas6 and its receptors in adipose tissue found that Axl is only expressed in pre-adipocytes, while Gas6, Mer, and Tyro3 are expressed in both pre-adipocytes and mature adipocytes [201]. Axl was found to be downregulated upon adipocyte differentiation, supporting a role in maintaining an undifferentiated state, much like in other cell types discussed [202]. Small molecule inhibition of Axl impairs pre-adipocyte differentiation, consistent with the decrease in weight gain of mice on a high fat diet relative to untreated mice [203]. However, another study found that *Axl* deficiency does not affect adipogenesis, where Tyro3 and Mer may respond to Gas6 in order to compensate for Axl [204]. This somewhat supports the findings that circulating sAxl has no significant correlation with adiposity in adolescents, while Gas6 does [205]. Expression of Axl is increased in liposarcoma compared to both pre-adipocytes and differentiated adipocytes, and only one further study has found it to be a prognosticator of survival by univariate analysis [206, 207]. Considering the widely credited role of Axl in migration/metastasis of tumor cells, Axl may not indeed contribute significantly to liposarcoma, which is rarely metastatic.

### Skin

Axl becomes overexpressed in melanoma and squamous carcinomas, compared to normal cells of the epithelium [208, 209]. Probably the most significant consequence of Axl upregulation in carcinomas of the skin is increased migratory ability of cells. In melanoma, Axl is associated with NRAS mutations compared to BRAF mutations, and is inversely correlated with the expression of the microphthalmia-associated transcription factor (MITF) [210–213]. Correlation studies also reveal the association of Axl with cell motility, invasion, and interactions with the surrounding microenvironment, and treatment with R428, a selective Axl inhibitor, reduces migration and invasion of cells [213]. Knockdown of *Axl* has uncovered STAT3 signaling as a downstream modulator of cell migration [214]. Interestingly, melanomas harboring Tyro3 display a higher proliferation rate in more differentiated cells, and this is consistent with the roles of Tyro3 and Axl in prostate cancer metastasis, as cited previously [213]. The differential phenotypes governed by each of the two receptors may represent a molecular switch in the development of cancer. Another report found that Axl and Mer are expressed in a mutually exclusive manner, where Mer is associated with BRAF mutations and Axl is associated with NRAS mutations [215]. Accordingly, treatment of NRAS-mutant melanoma cell lines with an inhibitor targeting multiple kinases including Axl, leads to growth arrest and apoptosis [210]. This drug has no effect in BRAS-mutant cell lines which lack Axl expression. Furthermore, overexpression of Axl increases the migratory ability of cells and is associated with genetic markers of invasion, whereas Mer is associated with markers of cell proliferation but is not sufficient to induce proliferation [215]. Both *Axl* and *Mer* inversely correlate with the expression of MITF, although it has not been determined whether they are direct targets of the transcription factor [215]. The mutual expression of Axl and N-cadherin in a heterogeneous melanoma cell population also marks a more invasive phenotype, compared to expression of MITF and E-cadherin [212]. Together, this suggests the effective use of Axl as a molecular biomarker for MITF-lacking melanomas, in which cells are less differentiated and have higher migratory ability.

Axl is widely implicated in mechanisms of therapeutic resistance in lung cancer, and is potentially linked to therapeutic resistance in melanoma. When melanoma cells are hit with chemotherapy and become senescent, they are able to alter the composition of their secretome toward being pro-inflammatory, and this has tumorigenic effects on neighboring melanoma-initiating cells [216]. Upregulation of Axl is an example of a molecular change in melanoma-initiating cells as a response to the secretome of cells undergoing senescence [216]. In mutant *BRAF*-harboring melanoma cells that are resistant to MAPK inhibitors, MITF is downregulated whereas Axl and NFκB signaling is upregulated [217]. Inhibition of Axl is able to restore sensitivity of cells to RAF and MEK inhibitors, except to the inhibition of ERK.

Axl has similar roles in the progression of squamous cell carcinomas (SCC), where it is overexpressed and contributes to cellular migration and EMT. Axl has even been used as a marker of SCC in development of an *in vitro* 3D model of SCC [218]. Clinically, Axl expression correlates with poor prognosis and lymph node status of oral SCC patients, and Gas6 activation induces an EMT-like gene signature [219]. Alternatively, Axl can also exert anti-apoptotic effects in SCC after UV exposure, contributing to the growth of tumors through Akt and suppression of Bcl-2 family members [220]. As in melanoma, Axl may contribute to the resistance of SCC to chemotherapy. This is mainly due to its effects on EMT by disrupting cell-cell adhesion in cancer stem cells through Wnt and TGFβ signaling [27].

### CNS

The TAM receptors are important for CNS development, but have not been well characterized in cells specific to the PNS. However, Gas6 stimulation of Axl and Tyro3 has been shown to act as a mitogenic factor for Schwann cells with implications in schwannoma [26, 221].

Gas6 is widely expressed in the CNS beginning in the late embryonic stages, and this is in contrast with protein S expression [222]. Many processes in the brain are regulated by the cooperation of two or more TAM receptors [223]. TKO mice have significantly reduced neural stem cell (NSC) proliferation and differentiation [224]. Consistent with Tyro3 being the most widely expressed TAM receptor in the brain, much of Axl signaling is dependent upon it [225]. In the rat brain, the expression of Axl across tissues is slightly different from that of Tyro3 [40]. In GnRH neurons, Axl and Tyro3 are expressed in migrating cells, whereas Mer and Tyro3 are expressed after migration [44]. The GnRH promoter is negatively regulated by MEF-2 transcription factors which can be induced by Axl in migrating cells [226]. Axl/Tyro3 null mice also result in increased apoptotic rate of GnRH neurons, implicating a normal role in apoptotic protection [227]. Protection from serum starvation-induced apoptosis is through both the PI3K/Akt and ERK pathways [228]. Furthermore, nerve growth factor (NGF) can regulate the expression and localization of Tyro3 and Axl, contributing to induction of neuronal differentiation [229]. Differential expression of Axl and Mer may also be responsible for the differential activity of Gas6 in early- and late-phase maturation of chondrocytes in the growth plate [95]. Taken together, this may suggest a phenomenon in which the ubiquitous expression of Tyro3 in the CNS can be altered to drive cell- and time-specific processes dependent upon the presence of either Axl or Mer. Overexpression of *Tyro3* in Axl-expressing Rat2 cells leads to a significant increase in cell proliferation, but this effect is not seen upon *Mer* overexpression, supporting this idea [46].

Alternatively, Axl and Mer are able to cooperate to inhibit Gas6 signaling in multiple sclerosis (MS), where they are found in their soluble forms [30]. The potential combination of Axl-mediated survival and Mer-mediated clearance of debris is thus inhibited, and may contribute to the pathology of MS [30]. In astrocytes, the combined signaling of Axl and Mer may account for chemotherapy resistance in astrocytoma patients [230]. Inhibition of Axl and Mer increases apoptosis and autophagy, and decreases cell colony formation, whereas restoration of either one alone does not compensate for these effects [230]. Microglia are macrophages of the brain, and just as Axl and Mer mediate macrophage engulfment of debris, they contribute to microglial phagocytosis of apoptotic cells and suppression of the immune response in the presence of Gas6 [224, 231]. However, just loss of *Axl* can also be responsible for increased axonal damage by inhibiting the ability of microglia to clear debris from demyelination [232, 233].

Axl mediates processes in the brain similar to in other cellular contexts. Anti-apoptotic signaling initiated by Gas6/Axl in cerebral endothelial cells is important for protection against hypertonicity induced by mannitol during clinical opening of the blood brain barrier for drug delivery [234]. In oligodendrocyte development, apoptotic inhibition by Gas6/Axl signaling is demonstrated to be through the PI3K/Akt pathway, specifically by recruiting GRB2 and the p85 subunit of PI3K [235, 236]. Thus, Gas6 is a key growth factor in the CNS. However, overactivation of pro-survival signaling by Gas6/Axl is involved in glioma growth and poor prognosis [158, 237, 238]. Expression of Axl in gliomas is also responsible for migration and invasion of cells, and may also contribute to maintenance of the neural stem/progenitor population [237, 239, 240]. Enhancer of zeste homolog 2 (EZH2) is a transcription factor that upregulates *Axl* expression in gliomas, and inhibition of EZH2 reduces invasiveness [241]. Additionally, Axl and Gas6 are coexpressed in tumor vessels, implying a role in neovascularization or angiogenesis [158].

Alzheimer's disease (AD) is another consequence of neuronal damage, and its pathogenesis may be caused, in part, by a deficiency in vitamin K. It is hypothesized that this directly affects Axl signaling in neurons because of the dependence of Gas6 on vitamin K for its γ-carboxylation and function as a growth factor [242]. Since Gas6/Axl signaling has been shown to protect neurons against apoptosis, lack of vitamin K would lead to increased apoptosis and neuronal damage [228]. In fact, Axl has been found to be a potential marker of brain amyloid burden associated with AD [243, 244].

### Eye

Axl signaling has implications in normal and disease processes in the eye. Axl is expressed in normal rat and bovine lens, specifically in the proliferating or quiescent epithelial cells in the periphery, rather than in differentiated cells [245]. There, Axl is responsible for maintaining cell proliferation and survival through the PI3K/Akt pathway [245]. Using an apoptosis-specific microarray chip to identify gene expression in the postnatal mouse lens, *Axl* was found to be developmentally regulated, implicating a normal role in development [246]. Another screen-based study found low levels of *Axl* in the normal rat retina, but increased levels after injury [247]. This is consistent with the role of Axl in helping to mediate clearance of apoptotic cells, as phagocytosis of apoptotic cells in the retina occurs when cells become photodamaged. Phagocytosis of apoptotic cells by retinal pigment epithelium (RPE) occurs via the same mechanism as macrophages in the immune response. However, instead of Gas6, photoreceptor-specific Tulp1 can bridge apoptotic cells and RPE cells through any of the TAM receptors [90]. During oxidative stress, the anti-aging gene Klotho increases RPE phagocytosis by upregulating TAM receptor expression through cAMP/PKA/CREB activation [248]. However, Axl may only have a small or supporting role in RPE clearance of apoptotic cells, as another study found that phagocytosis of apoptotic cells is cell-specific, where only Mer is required in the retina [45].

In addition to maintaining normal processes, Axl is involved in diseases of the eye. Axl is upregulated with age in the lens, as detected in a model of age-onset cataract [249]. Oxidative stress is a contributor to age-related cataract, and *Axl* transcript levels are upregulated in the acute response to H_2_O_2_ treatment [250]. In uveal melanoma, Axl may be a key factor in maintaining the balance of proliferation, apoptosis, and angiogenic suppression needed for micrometastatic dormancy [251]. Gas6/Axl signaling decreases the angiogenic factor Cyr61, mildly protects cells from apoptosis, and increases cell proliferation [251]. Treatment of retinoblastoma cell lines with a therapeutic agent usually used for macular degeneration is able to inhibit growth and proliferation, and this is associated with decreased Axl expression [252]. As mentioned previously, YAP regulates the transcription of *Axl*, and treatment with this therapeutic downregulates YAP [252].

### Thyroid

Axl is normally expressed at none or very low levels in the thyroid, but its overexpression contributes to thyroid cancer [253, 254]. *Gas6* mRNA is also expressed in thyroid cancer cells where it has a slight mitogenic effect, and thus is considered a growth factor for Axl-expressing thyroid carcinoma cells [254]. Thyroid carcinomas induced by exposure to radiation show increased levels of both Gas6 and Axl with reduced apoptosis, implying an autocrine activation of Axl [255]. Axl is faintly expressed in human thyroid adenomas and is highly expressed in carcinomas, with no expression in normal thyroid tissue [253]. However, in contrast to breast cancer, Axl does not correlate with lymph node metastasis [253]. CXCR4/SDF-1 signaling transcriptionally regulates both *Tyro3* and *Axl*, but silencing of just *Axl* leads to a decrease in cancer cell invasion, increased apoptosis, and inhibition of tumor formation due to inhibition of angiogenesis [256]. Expression of Axl along the spectrum of differentiated thyroid cancers is equal, indicating it is part of a cancer-initiating event, rather than a consequence [256]. Alternatively, one study found that Axl expression is decreased in tissue associated with malignancy, but elevated in the serum of patients with extrathyroidal invasion and lymph node metastasis [257]. These conflicting results may be attributed to the method of detection, as presence of Axl in the serum is most likely a result of the cleaved, soluble form. This may coincide with decreased detection of Axl in the tissues.

### Colon

The oncogenic properties of Axl have implications for the progression of colon carcinomas, as it is weakly expressed in the normal colon [258]. Axl is overexpressed in malignant cells, specifically in peritoneal metastases which represent the most aggressive form of colon cancer [258, 259]. MZF1 upregulates *Axl* and both genes positively correlate with colorectal tumors, where MZF1 induces invasion and metastasis [65]. Structurally similar to YAP, TAZ is also a transcriptional regulator of *Axl*, and in colorectal cancers correlates with shorter survival [260]. Therefore, both TAZ target genes *Axl* and *CTGF* are also predictors of patient survival, where patients expressing all three genes have worse prognosis than those that express just two or one [260]. Knockdown of TAZ decreases Axl expression, and leads to decreased proliferation, reduction in non-adherent colony formation, and decreased tumorigenesis [260]. Furthermore, genes associated with EMT are also overexpressed in patients expressing TAZ, Axl, and CTGF, in support of the more aggressive status of Axl-expressing tumors [260]. Consistent with this finding, knockdown of CXCR4 and Axl reduces invasion of colon cancer cells [261]. As in thyroid cancer, CXCR4/SDF-1 signaling leads to the transcriptional regulation of *Axl*, which has implications in metastasis [261]. Axl also contributes to chemoresistance in colon cancer, as it most prominently does in lung cancer [261]. The metastatic and invasive properties of Axl contribute to its role in resistance, as micrometastases lay dormant and thus are not susceptible to standard chemotherapy. Axl phosphorylation is increased in invasive colon cancer cell lines, while RTKs involved in maintaining epithelial status are downregulated [262]. In this setting, inhibition of Axl leads to decreased STAT3, SFK, and Akt activities, consequently reducing cellular migration rate [262]. This relates to therapeutic resistance in that treatment of non-invasive parental colon cancer cell lines with an adjuvant leads to an increase in migration and invasion, but this increase is eliminated upon silencing of *Axl* [262]. In gastrointestinal stromal tumors, the upregulation of Axl in resistance to imatinib mesylate acts as a molecular switch from c-KIT expressing tumors [263].

These findings suggest that Axl may be a prognostic biomarker and therapeutic target in colorectal cancer. Targeting Axl in colorectal cancer may need to occur after or in combination with chemotherapy. A multikinase inhibitor to Axl, MET, and FGFR is only effective in preventing tumor growth after or in combination with a VEGF antagonist, and either treatment alone is not as effective [264]. Thus, Axl may play a secondary role in the dependency of the tumor [264]. It is important to note that studies of TAM receptor inhibition in the colon have revealed potential adverse effects of systemic therapies targeting TAM receptors, specifically Axl and Mer [91]. In addition to their oncogenic roles, Axl and Mer function to limit the inflammatory response in dendritic cells and macrophages, as previously explained. Since inflammation often sets the stage for cancer initiation, as in ulcerative colitis patients, Axl and Mer acquire a protective role [265]. Thus, targeted therapy against Axl and Mer in other cancers may compromise their ability to limit colonic inflammation, increasing the risk for inflammation-associated colorectal cancer [91]. It then becomes desirable to develop tumor-specific approaches to target Axl and Mer signaling.

### Liver

Tissue repair and regeneration as well as the hepatic immune system are essential aspects of normal liver homeostasis. Gas6/Axl signaling has important implications in these normal processes, as well as those involved in disease.

Gas6 and Axl are mainly expressed in oval cells of the liver, and not in hepatocytes [266]. Oval cells are precursors which differentiate and proliferate upon hepatic injury, and display some of the same surface markers as hematopoietic stem cells [267]. In these cells, Gas6 acts as a survival factor that protects against apoptosis during experimental serum deprivation, suggesting its role in maintaining the population of precursor cells during regeneration [266]. Oval cells are the secondary response to hepatic injury, in the situation where hepatic stellate cells (HSCs) are unable to proliferate. HSCs are mature cells that are responsible for the liver's regenerative ability, and which accumulate at the site of injury and transform into cytokine-secreting myofibroblasts [268]. Just as Gas6 is a survival factor for oval cells, it is upregulated and has the same function in HSCs after treatment with CCl_4_, an injury-mimicking agent known to induce HSC regeneration [269]. Axl is also expressed in HSCs, and signals through the PI3K/Akt and NFκB pathways to protect against apoptosis [269]. Axl and Gas6 are expressed in macrophages near the site of injury, which may aid in the uptake of apoptotic cells that occurs before regeneration [269]. Not surprisingly, injection of CCl_4_ into *Gas6*-deficient mice leads to impaired liver repair and decreased cytokine synthesis, through SOCS1 induction by Axl in a Gas6-independent manner [5]. Further studies with *Gas6*−/− mice have implicated the Gas6/Axl pathway in the progression of steatohepatitis and fibrosis [270]. Consistent with the function of Axl in limiting the immune response in the liver, knockout of all three TAM receptors leads to dramatic liver damage due to inflammation, demonstrating their essential role in the immune tolerance of the liver [271].

Axl is found to be upregulated in hepatocellular carcinoma (HCC) tumors compared to normal hepatocytes, but these observations are variable, and Axl seems to be more associated with lymph node metastasis [272–276]. Knockdown of *Axl* in metastatic HCC cells inhibits their metastasis to lymph nodes *in vivo*, which may be due to the Gas6/Axl mediated decrease in Cyr61, an angiogenic factor also regulated by Gas6/Axl in the eye [277]. Gas6 activation of Axl is able to induce the EMT-associated transcription factor Slug, but not Snail, Twist, or Zeb1/2 in HCC cells [275]. This is through activation of ERK, and leads to invasion and migration of cells [275]. Interestingly, this study did not detect increased levels of Axl or Gas6 in HCCs compared to normal liver tissue, implying that the migratory effects downstream of Axl are not due to its activation by Gas6, but by activation in a ligand-independent manner [275]. Another study found Axl modulation of the PI3K/Akt pathway to be responsible for the enhanced migratory ability of a metastatic HCC cell line, and that Axl expression correlates with pathological features of HCC in patients including lymph node metastasis [274].

The regulation of Axl in HCC has been explored, and is similar to in other cell contexts. As in lung cancer, YAP has oncogenic implications in the liver and requires Axl for migration, invasion, proliferation, and survival of YAP-transformed HCC cells [68]. Oncogenic signaling by YAP is partly due to Axl activation of ERK1/2 signaling [68]. The deglycosylation of Axl by tunicamycin was shown to inhibit the proliferation and lymph node metastasis of a metastatic HCC line [278]. However, the global inhibition of glycoprotein synthesis induced by tunicamycin may also induce off-target effects partly responsible for the observed effects of the study. Loss of miR-122, a microRNA involved in the maintenance of hepatic function in mice, leads to increased expression of *Axl*, which was found to be a target of miR-122 in HCC [279]. miR-122 deficiency in the liver is associated with tumor formation, and thus the subsequent upregulation of *Axl* may be responsible for miR-122-associated tumorigenesis [279].

## Clinical implications for Axl

After examining the role of Axl in each cell type, it is evident that therapeutic targeting of Axl would be beneficial in disease, specifically in cancer. It is also evident that its role in each cell type is distinct and depends on other surrounding factors. Thus, development of Axl-targeted therapeutics for specific cancers requires knowledge about what Axl regulates, what regulates Axl, and what interacts with Axl in each context. The conflicting roles of Axl/Mer in limiting the immune response while also promoting tumorigenesis has already been implicated as a therapeutic obstacle, and further supports the necessity for cell- or tumor-specific treatments [91].

Significant progress has been made in the development of Axl inhibitors since the late discovery of Axl in 1991. One of the most frequently used and most potent Axl inhibitors in the laboratory setting, BGB324 (formerly R428), is the first small molecule inhibitor of Axl to enter clinical trials. It is currently in phase I trials, and its clinical response is to be assessed in AML and NSCLC patients. Other Axl inhibitors in development and clinical trials are listed in Table 3, and are further described in Feneyrolles, et al. [280]. It is unlikely that selective inhibition of Axl will be an effective monotherapy in cancer. Taken together, the literature has revealed an apparent passenger, rather than driver, role for Axl in the progression and resistance of tumors. Selective inhibition of Axl may be effective in tumors which have already, or are predicted to become, resistant to other therapies.

**Table 3:** Axl inhibitors in pre-clinical and clinical stages

Name	Target(s)	Development Status	Reference (with Axl)
SGI-7079	Axl, FLT3, Mer, MET, TrkA/B, Ret, Yes, Jak2, VEGFR2, JNK3 Abl	Pre-clinical	[146]
GL21.T	Axl	Pre-clinical	[283]
NPS-1034	MET, Axl	Pre-clinical	[155]
TP-0903	Axl	Pre-clinical	(Tolero website)
BGB324 (R428)	Axl	Phase I	[122]
SU11248 (Sunitinib)	FLT3, VEGFR2, KIT, Axl	Phase I	[284]
S49076	MET, Axl/Mer, FGFR1/2/3	Phase I	[264]
LY2801653	Ron, MET, MST1R, FLT3, Axl, Mer, TEK, ROS1, DDR1/2, MKNK1/2	Phase I	[285]
BMS777607	MET, Ron, FLT3, Axl, Mer, Tyro3	Phase I	[286]
MGCD265	MET, Ron, VEGFR1/2/3, Tie-2, Axl	Phase II	[280]
SKI606 (Bosutinib)	Axl, SRC/Abl	Phase II	[117]
MP470 (Amuvatinib)	KIT, PDGFRα, Axl	Phase II	[263]
GSK1363089 (Foretinib)	Axl, MET, VEGFR2, Ron, Tie2, KIT	Phase II	[118]
XL184 (Cabozantinib)	MET, VEGFR2, RET, KIT, Axl, FLT3	Phase III	[287]

## Concluding remarks

Axl has emerged as a critical player in the immune response and in cancer. Axl is usually expressed at lower levels in normal tissue compared to in the disease state, indicating an oncogenic role for the receptor. There are several reasons to suggest that Axl functions to support processes which have already undergone an initiating step: (1) the lack of known activating mutations, (2) the lack of its ability to transform cells, as demonstrated in its initial characterization, (3) the lack of a major role in development, and (4) its overexpression/overactivation in therapeutic resistance.

Axl signals through and amplifies existing pathways rather than through any “Axl-specific” pathway. Axl is cooperative in nature and future research will hopefully identify functional relationships with its other family members, Tyro3 and Mer. It is clear that the consequence of Axl signaling varies between cell type and depends on the presence of other signaling molecules, so it is crucial to establish an understanding of this within each context.
